# Characteristics of Clinical Response in Patients With Chronic Myeloid Leukemia With an *ABL1* In‐Frame Exon 4 Deletion: A Multicenter Retrospective Study

**DOI:** 10.1002/jha2.70361

**Published:** 2026-07-27

**Authors:** Yuto Kaneda, Nobuhiro Kanemura, Yoshikazu Ikoma, Takuro Matsumoto, Nobuhiko Nakamura, Hiroshi Nakamura, Senji Kasahara, Kenji Fukuno, Yutaka Tsukune, Tomoiku Takaku, Masahito Shimizu

**Affiliations:** ^1^ Department of Hematology and Infectious Disease Gifu University Hospital Gifu Japan; ^2^ Division of Cellular Medical Sciences Department of Biochemistry and Molecular Biology Kobe University Graduate School of Medicine Kobe Japan; ^3^ Department of Hematology Gifu Municipal Hospital Gifu Japan; ^4^ Department of Hematology Japanese Red Cross Takayama Hospital Gifu Japan; ^5^ Department of Hematology Juntendo University School of Medicine Tokyo Japan; ^6^ Department of Hematology Saitama Medical University Saitama Japan

**Keywords:** *BCR::ABL1*, chronic myeloid leukemia, Exon 4 deletion, splicing variant, tyrosine kinase inhibitor

## Abstract

**Introduction:**

In‐frame Exon 4 deletion (p.L184_K274del) is a rare *ABL1* splicing variant in chronic myeloid leukemia (CML), the clinical significance of which remains unclear.

**Methods:**

We retrospectively analyzed seven chronic‐phase CML patients with this variant, identified by Sanger sequencing during routine monitoring of atypically slow molecular responses.

**Results:**

All patients achieved hematologic responses; molecular responses, although attained, were often delayed. No patient progressed to advanced phase disease during a median follow‐up of 39.0 months.

**Conclusion:**

Detection of this kinase‐inactive variant should not, in itself, be regarded as a definitive resistance mechanism. Larger collaborative studies using quantitative methodologies are needed.

**Trial Registration:**

The authors have confirmed clinical trial registration is not needed for this submission.

## 1 Introduction

Patients with chronic myeloid leukemia (CML) present a specific genetic abnormality, the Philadelphia chromosome, resulting from *t* (9;22) translocation [1], which leads to formation of the *BCR::ABL1* abnormal fusion gene. The BCR–ABL1 protein encoded by this fusion gene possesses persistent tyrosine kinase activity, driving the pathogenesis of CML. Treatment with tyrosine kinase inhibitors (TKIs), as well as a first‐in‐class specifically targeting the BCR–ABL myristoyl pocket (STAMP) inhibitor, has dramatically improved the prognosis of CML patients [2]. Following the introduction of imatinib, a TKI, the development of second‐generation (nilotinib, dasatinib, and bosutinib) and third‐generation TKIs (ponatinib) and the STAMP inhibitor asciminib has led to their clinical application in Japan, enabling more CML patients to achieve a deep molecular response. On the other hand, mutations in the *ABL1* kinase domain are a major factor inducing treatment resistance through structural changes in the TKI binding site and increased tyrosine kinase activity [3]. Although point mutations predominate [4], rare *BCR::ABL1* transcript variants generated by aberrant splicing have been described, including Exon 7 skipping [5], exon 8/9 35‐bp insertion [6], and an in‐frame deletion at p.L184_K274 in Exon 4 [7]. Among these, Exon 7 skipping is well established as a non‐pathogenic splicing event with no demonstrable impact on TKI response, and Exon 4 deletion likely shares a similar splicing mechanism. The biological significance of Exon 4 deletion in the context of clinical TKI response, however, remains uncharacterized. In addition, rare *BCR::ABL1* transcript variants, such as those with *ABL1* in‐frame Exon 4 deletion, present distinct clinical challenges, particularly due to limited data on their impact on treatment response to second‐ and third‐generation TKIs and STAMP inhibitors [8, 9]. Here, we report the clinical course and molecular responses of seven CML patients with the *ABL1* in‐frame Exon 4 deletion.

## 2 Materials and Methods

We conducted a multicenter retrospective study to investigate molecular response patterns in this rare CML subset. We analyzed seven chronic phase CML patients from multiple centers in Japan, who harbored the confirmed p.L184_K274del (c.550_822del273) in‐frame deletion. At diagnosis, *BCR::ABL1* transcripts were detected by a standard qRT–PCR assay designed to detect the major transcripts (e13a2 and e14a2) in a single reaction. The Exon 4 deletion was identified by Sanger sequencing performed at an external commercial reference laboratory, prompted by a slower‐than‐expected reduction in BCR–ABLIS transcript levels during follow‐up. We investigated the clinical characteristics, treatment course, and changes in international scale (IS) transcript levels for each case. Disease phases were defined according to the ELN 2020 criteria [10]. This study was approved by the Institutional Review Boards of all participating centers (approval number: 2023–114), and was conducted in accordance with the Declaration of Helsinki.

## 3 Results

Median patient age at diagnosis was 44 years (range: 25–74 years), and four of the seven cases were female. All patients were confirmed to have no other concurrent *ABL1* kinase domain mutations coexisting with p.L184_K274del. The major *BCR::ABL1* transcript was detected in all patients. Median white blood cell count at diagnosis was 225.8 × 10^3^/µL (range: 19.3–472.3), and platelet count was 478.0 × 10^3^/µL (range: 137.0–1458.0). Among the seven cases, a high‐risk Hasford score was observed in four patients, a high‐risk Sokal score in five patients, a high‐risk European Treatment and Outcome Study (EUTOS) scores in four patients, and a high‐risk EUTOS long‐term survival (ELTS) scores in three of the seven cases. No additional high‐risk chromosomal abnormalities (ACAs) were detected (Table 1, Table S1).

**TABLE 1:** Baseline characteristics of patients with *ABL1* in‐frame Exon 4 deletion.

Variables	Total cohort (*n* = 7)
Age, years, median (range)	44 (25–74)
Sex	
Female, *n* (%)	4 (57.1)
Male, *n* (%)	3 (42.9)
WBC (× 10^3^/µL), median (range)	225.80 (19.30–472.34)
Blasts (%), median (range)	3.0 (0.0–9.0)
Basophils (%), median (range)	5.0 (1.5–9.0)
Eosinophils (%), median (range)	2.5 (0.5–7.0)
Hemoglobin (g/dL), median (range)	11.0 (7.3–15.1)
Platelets (× 10^3^/µL), median (range)	478.0 (137.0–1458.0)
High‐risk ACA	0 (0.0)
Spleen size (cm), median (range)	14.7 (9.0–19.5)
Hasford point, median (range)	1694.2 (1017.1–3077.6)
Hasford score	
High, *n* (%)	4 (57.1)
Intermediate, *n* (%)	2 (28.6)
Low, *n* (%)	0 (0.0)
Nonevaluable, *n* (%)	1 (14.3)
Sokal point, median (range)	1.36 (1.08–5.25)
Sokal score	
High, *n* (%)	5 (71.4)
Intermediate, *n* (%)	1 (14.2)
Low, *n* (%)	0 (0.0)
Nonevaluable, *n* (%)	1 (14.2)
EUTOS point, median (range)	100.4 (64.0–140.2)
EUTOS score	
High, *n* (%)	4 (57.1)
Intermediate, *n* (%)	0 (0.0)
Low, *n* (%)	2 (28.5)
Nonevaluable, *n* (%)	1 (14.2)
ELTS point, median (range)	2.24 (1.33–2.56)
ELTS score	
High, *n* (%)	3 (42.9)
Intermediate, *n* (%)	2 (28.5)
Low, *n* (%)	1 (14.2)
Nonevaluable, *n* (%)	1 (14.2)

Abbreviations: ACA, additional cytogenetic abnormalities; ELTS, EUTOS long‐term survival score; EUTOS, European Treatment and Outcome Study; WBC, white blood cell.

**FIGURE 1:**
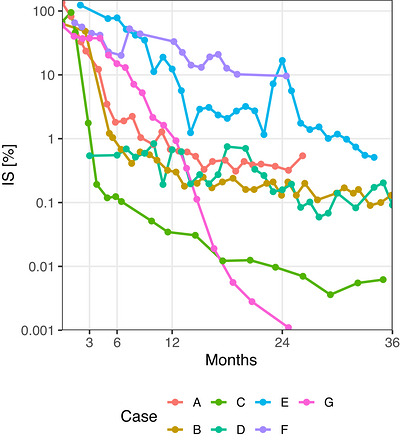
Comparison of the clinical course of the international scale of transcription levels in seven cases with *ABL1* in‐frame Exon 4 deletion. IS: international scale.

The median observation period was 39.0 months (range: 24.7–82.8 months). All cases achieved a complete hematologic response (CHR) with the initial TKI therapy, although molecular responses were often delayed (Figure 1). Median BCR–ABL^IS^ levels were 37.4% (range: 0.54%–76.0%) at 3 months, 1.81% (range: 0.12%–78.1%) at 6 months, 0.66% (range: 0.03%–33.2%) at 12 months, and 0.14% (range: 0.0028%–9.63%) at 24 months. Only one case achieved a major molecular response (MMR; BCR–ABL1^IS^ ≤ 0.1%) at 12 months. Overall, an MMR was achieved in four of the seven cases, with a median time to achievement of 21.4 months (range: 9.93–34.1). A deeper molecular response (DMR; BCR–ABL1^IS^ ≤ 0.01%) was achieved in two of the seven patients, with a median time to achievement of 21.1 months (range: 18.9–23.6). Initial TKI therapy included nilotinib (four cases), dasatinib (two cases), and bosutinib (one case), with a median of two treatment lines (range: 1–5) (Table S2). Two of seven cases continued initial TKI therapy. In the later lines of treatment, ponatinib was used in four cases and asciminib in two cases (Figure S1). At the time of this publication, all patients remain on ongoing therapy, without progressing to an accelerated phase or blast phase of CML.

## 4 Discussion

In CML patients with the in‐frame Exon 4 deletion, the response that is achievable but is sometimes delayed contrasts with the rapid resistance frequently seen in patients with point mutations in the *ABL1* kinase domain. The slow clinical course without acute transformation, despite the presence of an *ABL1* alteration, is consistent with the catalytically inactive nature of the p.L184_K274del variant reported by Sherbenou et al. [7], rather than with an aggressive kinase‐driven process. The slow clinical course without acute transformation despite the presence of an *ABL1* mutation suggests reduced tyrosine kinase activity of the BCR–ABL1 protein due to kinase dysfunction associated with the p.L184_K274del mutant [7]. On the other hand, only one case in this study achieved MMR at 12 months, contrasting with the typical response rates observed in CML populations treated with TKIs and STAMP inhibitors. Nevertheless, deep and sustained responses were ultimately achievable in some patients.

The kinase‐inactivating Exon 4 deletion abolishes the proliferative advantage of variant‐bearing cells, which therefore behave as non‐leukemic bystanders that become relatively enriched after TKI therapy; their persistent detection by Sanger sequencing would then reflect a non‐proliferating variant‐bearing population rather than true MRD of the proliferative leukemic clone, and the apparent delay in molecular response could partly reflect this persistence rather than ongoing kinase‐driven leukemia. This interpretation is consistent with the kinase‐inactivity data reported by Sherbenou et al. [7], with the absence of any other detectable *ABL1* kinase domain mutation, and with the favorable responses observed in all patients, making it unlikely that the Exon 4 deletion itself confers obvious resistance. Indeed, *BCR::ABL1* splicing variants such as the exon 8/9 35‐bp insertion have similarly been shown to be functionally inactive, and both this insertion variant and the Exon 4 p.L184_K274 deletion have been reported to persist under TKI therapy as loss‐of‐function transcripts [6, 8].

Regarding individual case variations in TKI responsiveness, non‐*BCR::ABL*‐dependent factors should also be considered. These include activation of intracellular signaling pathways by driver gene abnormalities other than *BCR::ABL1*, DNA repair defects or genomic instability, clonal evolution via epigenetic mechanisms, failure of immunological antitumor effects in the bone marrow microenvironment, and the impact of ATP‐binding cassette transporters on TKI concentration, all of which have been reported as potential mechanisms of variations in TKI responsiveness [11].

Clinically, since Sherbenou et al. demonstrated that the p.L184_K274 deletion lacks most of the kinase domain, including the P‐loop, is non‐oncogenic and catalytically inactive, and confers increased rather than decreased imatinib sensitivity in vitro [7], our findings should not be interpreted as evidence that the variant itself drives TKI resistance; as with Exon 7 skipping [5], it may instead represent a non‐pathogenic splicing product. Accordingly, premature treatment modification on the basis of this variant alone is not warranted when the molecular response continues to deepen, and treatment decisions should instead be guided by standard molecular response milestones and by quantitative monitoring of functional *BCR::ABL1* transcripts.

This study has several limitations. First, we could not formally demonstrate that the deletion is localized to the *BCR::ABL1* fusion transcript rather than the wild‐type *ABL1* allele, although no Exon 4 deletion has been reported in healthy individuals. Clinicians should, therefore, not interpret the presence of this variant as definitive evidence of a resistance mechanism. Second, since the analysis was performed only once per patient at an external reference laboratory, neither technical reproducibility, reproducibility across timepoints, nor allele‐burden quantification (e.g., by digital PCR or deep sequencing) could be assessed. Third, detection by Sanger sequencing typically requires a residual CML cell burden of ≥ 10%, the exact PCR primer configuration was unavailable, and the frequency of this variant could not be estimated from this retrospectively assembled cohort. Finally, since *ABL1* mutation testing was not routinely performed on diagnostic samples, we cannot determine whether the deletion was already present at the time of diagnosis or appeared during the course of TKI therapy.

Future prospective studies incorporating allele‐specific quantitative sequencing, duplicate and serial‐timepoint analysis, and diagnostic‐sample screening are required to clarify the biological and clinical significance of this variant.

## 5 Conclusion

In this multicenter retrospective study, CML patients harboring the *ABL1* Exon 4 in‐frame deletion ultimately achieved molecular responses with TKI therapy, although often with a delay, and none progressed to an advanced phase. Given the established kinase inactivity of this variant and the methodological limitations described, detection of the Exon 4 deletion should not by itself prompt treatment modification, and standard molecular response milestones should remain the primary guide for clinical decisions. Larger collaborative studies with quantitative methodologies are essential for clarifying the biological and clinical significance of *ABL1* Exon 4 in‐frame deletion in CML patients.

## Author Contributions

All authors contributed to the study conception and design. Material preparation and data collection were performed by Y.K., N.K., Y.I., T.M., H.N., S.K., K.F., Y.T. and T.T. The first draft of the manuscript was written by Y.K. Supervision was provided by T.T. and M.S. All authors commented on previous versions of the manuscript and approved the final manuscript.

## Funding

The authors have nothing to report.

## Ethics Statement

This study was performed in line with the Principles of the Declaration of Helsinki. Approval was granted by the Medical Review Board of Gifu University Graduate School of Medicine (Approval number: 2023–114). This multicenter study was approved via a Centralized IRB Review Process, and Institutional Permission was obtained at each participating site.

## Consent

The requirement for informed consent was waived by the ethics committee due to the retrospective nature of the study and the use of anonymized patient data.

## Conflicts of Interest

Yuto Kaneda has received honoraria from AbbVie GK and Novartis. Yoshikazu Ikoma has received honoraria from Meiji Seika Pharma Co. Ltd., AbbVie GK, Astellas Pharma Inc., Nippon Shinyaku Co. Ltd., Chugai Pharmaceutical Co. Ltd., and MSD K.K. Nobuhiko Nakamura has received honoraria from Chugai Pharmaceutical Co. Ltd., BeiOne Medicines LLC, AbbVie GK, Kyowa Kirin Co. Ltd., Sanofi, Daiichi Sankyo Co. Ltd., Nippon Shinyaku Co. Ltd., Takeda Pharmaceutical Co. Ltd., and Janssen Pharmaceutical K.K. The other authors declare no conflicts of interest.

## Supporting information




**Supporting Information file**: jha270361‐sup‐0001‐SuppMat.pdf

## Data Availability

The data that support the findings of this study are not publicly available due to their containing information that could compromise the privacy of research participants, but are available from the corresponding author upon reasonable request.
