# Supplementary material for: Characteristics of Clinical Response in Patients With Chronic Myeloid Leukemia With an ABL1 In‐Frame Exon 4 Deletion: A Multicenter Retrospective Study
Source: EJHaem. 2026 Jul 27;7(4):e70361. doi: 10.1002/jha2.70361 (PMC13404248; doi:10.1002/jha2.70361)
Supplement: Supplementary file 1 — Supporting Information file: jha270361‐sup‐0001‐SuppMat.pdf [file JHA2-7-e70361-s001.pdf]

Supplementary Table 1. Individual baseline characteristics of the seven patients with ABL1 in-frame exon 4 deletion evaluated in this study

Case	Disease	Age	Sex	ABL1 mutation	WBC ($\times 10^3/\mu L$)	Blasts (%)	Basophils (%)	Eosinophils (%)	Hemoglobin (g/dL)	Platelets ($\times 10^4/\mu L$)	High-risk ACA	Spleen size (cm)	Hasford point	Hasford score	Sokal point	Sokal score	EUTOS point	EUTOS score	ELTS point	ELTS score
A	CML-CP	42	F	p.L184_K274del	225.8	5	9	7	11	92.3	None	19.3	1595.6	High	2.38	High	140.2	High	2.33	High
B	CML-CP	52	M	p.L184_K274del	160.48	0	6	2.5	8.3	28.4	None	19.5	1792.8	High	1.29	High	120	High	2.33	High
C	CML-CP	29	M	p.L184_K274del	233.4	3	3	0.5	7.5	25	None	14.7	1017.1	Intermediate	1.08	Intermediate	79.8	Low	2.1	Intermediate
D	CML-CP	74	M	p.L184_K274del	19.3	0	1.5	2.5	15.1	13.7	None	NA	NA	NA	NA	NA	NA	NA	NA	NA
E	CML-CP	44	F	p.L184_K274del	314.5	1.4	4	2	11.7	95.9	None	9	1841.9	High	1.28	High	64	Low	1.33	Low
F	CML-CP	25	F	p.L184_K274del	472.34	6	5	3.5	7.3	47.8	None	14.7	1316	Intermediate	1.44	High	93.8	High	2.16	Intermediate
G	CML-CP	62	F	p.L184_K274del	99.37	9	9	3	11	145.8	None	11	3077.6	High	5.25	High	107	High	2.56	High

Abbreviations: ACA: Additional cytogenetic abnormalities; CML-CP: Chronic myeloid leukemia in chronic phase; EUTOS: European treatment and outcome study; ELTS: EUTOS long-term survival score; NA: Not available; WBC: White blood cell.

Supplementary Table 2. Summary of therapeutic outcomes for patients with ABL1 in-frame exon 4 deletion

Variable	All patients (n=7)
Initial TKI	
Dasatinib – *n* (%)	2 (28.5)
Nilotinib – *n* (%)	4 (57.1)
Bosutinib – *n* (%)	1 (14.2)
Treatment lines – median (range)	2 (1 - 5)
Achievement of CHR – *n* (%)	7 (100)
International Scale	
3 months (%) – median (range)	37.4 (0.54 - 76.0)
6 months (%) – median (range)	1.81 (0.12 - 78.1)
12 months (%) – median (range)	0.66 (0.03 - 33.2)
24 months (%) – median (range)	0.14 (0.0028 - 9.63)
3 month IS ≤ 10% – *n* (%)	2 (28.5)
6 month IS ≤ 1% – *n* (%)	2 (28.5)
12 month IS ≤ 0.1% – *n* (%)	1 (14.3)
24 month IS ≤ 0.01% – *n* (%)	2 (28.5)
Anytime IS ≤ 0.1% – *n* (%)	4 (57.1)
Anytime IS ≤ 0.01% – *n* (%)	2 (28.5)
Time to achieve IS ≤ 0.1% – median (range)	21.4 (9.93 - 34.1)
Time to achieve IS ≤ 0.01% – median (range)	21.1 (18.9 - 23.6)

Abbreviations: CHR: Complete hematologic response; IS: International Scale; TKI: Tyrosine kinase inhibitor.

Figure Legend

Supplementary Figure 1. Summary of the clinical course of patients with ABL1 in-frame exon 4 deletion based on the treatment received

Abbreviations: IS: International Scale.

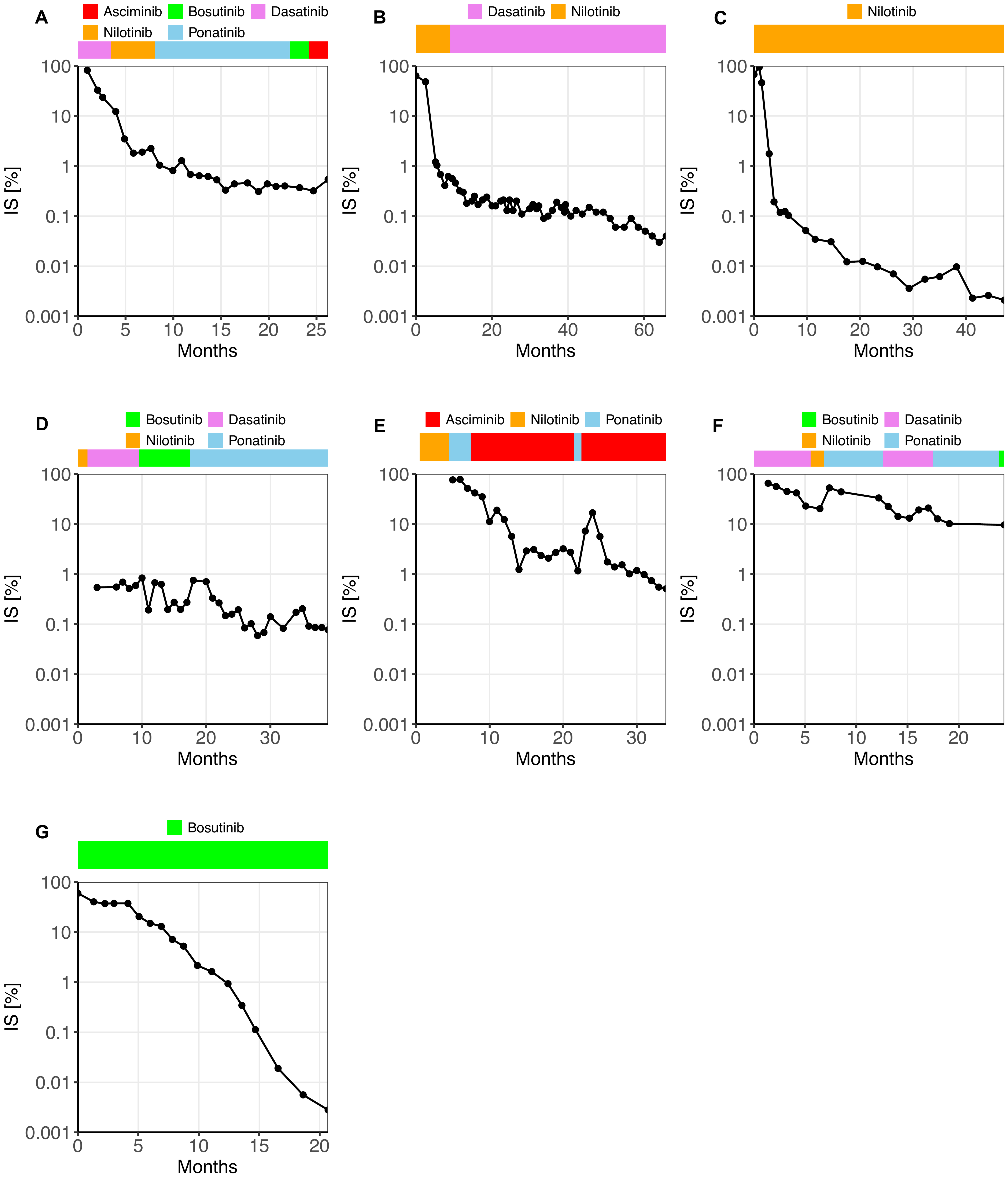

Supplementary Figure 1
